# Outcome of patients with curative-intent treatment for primary pulmonary sarcoma: Results from an international multicenter retrospective study

**DOI:** 10.1016/j.xjon.2025.06.024

**Published:** 2025-09-26

**Authors:** Stephane Collaud, Theresa Stork, Dagmar Adamkova, Clemens Aigner, Ivan Bravio, Antonella Brunello, Luigi Cerbone, Hugo Clermidy, Lore De Cock, Silvia Gasperoni, Nicolas Girard, Anna Mariuk-Jarema, Rolf Lefering, Enrico Melis, Gloria Marquina, Filomena Mazzeo, Iurii Mykoliuk, Maria A. Pantaleo, Nicolas Penel, Hans-Ulrich Schildhaus, Sabino Strippoli, Bruno Vincenzi, Sarah Watson, Jean-Yves Blay, Sebastian Bauer

**Affiliations:** a Department of Thoracic Surgery, University of Witten/Herdecke, Kliniken der Stadt Koeln, Lung Clinic, Cologne, Germany; b Department of Comprehensive Cancer Care, Masaryk Memorial Cancer Institute and Faculty of Medicine, Masaryk University, Brno, Czech Republic; c Department of Thoracic Surgery, Medical University of Vienna, Vienna, Austria; d Department of Thoracic Surgery, Francisco Gentil Portuguese Institute of Oncology, Lisboa, Portugal; e Medical Oncology 1 Unit, Istituto Oncologico Veneto IOV-IRCCS, Padova, Italy; f SSD Mesotelioma, melanoma e tumori rari, Azienda Ospedaliera Universitaria SS Antonio e Biagio e Cesare Arrigo, Alessandria, Italy; g Department of Thoracic Surgery, Louis Pradel Hospital, Hospices Civils de Lyon, Lung and Heart-Lung Transplantation, Lyon, France; h Department of General Medical Oncology, University Hospitals Leuven, Leuven Cancer Institute, Leuven, Belgium; i Laboratory of Experimental Oncology, KU Leuven, Leuven Cancer Institute, Leuven, Belgium; j Department of Oncology, Clinical Oncology Unit, University Hospital Careggi, Firenze, Italy; k Institut du Thorax Curie-Montsouris, Institut Curie, Paris, France; l Department of Soft Tissue/Bone Sarcoma and Melanoma Maria Sklodowska-Curie National Research Institute of Oncology, Warsaw, Poland; m Institute for Research in Operative Medicine (IFOM), University Witten/Herdecke, Cologne, Germany; n Thoracic Surgery Unit, IRCCS National Cancer Institute Regina Elena, Rome, Italy; o Department of Medical Oncology, Department of Medicine, Hospital Clínico San Carlos, School of Medicine, Instituto de Investigación Sanitaria (IdISSC), EURACAN Referral Centre, Universidad Complotense de Madrid (UCM), Madrid, Spain; p Department of Clinical Oncology, Cliniques Universitaires Saint Luc-Institut Roi Albert II, Université Catholique de Louvain, Brussels, Belgium; q Division of Thoracic Surgery and Hyperbaric Surgery, Department of Surgery, Medical University of Graz, Graz, Austria; r Oncology Unit, IRCCS AOUBO, Bologna, Italy; s Department of Medical Oncology, Centre Oscar Lambret, University of Lille, Lille, France; t Discovery Life Sciences Biomarker GmbH und Pathologie Nordhessen, Kassel, Deutschland; u Unità Operativa Tumori Rari e Melanoma, I.R.C.C.S.Istituto Tumori “Giovanni Paolo II”, Bari, Italy; v Operative Research Unit of Medical Oncology, Fondazione Policlinico Universitario Campus Bio-Medico, Roma, Italy; w Department of Medicine and Surgery, Università Campus Bio-Medico di Roma, Roma, Italy; x Medical Oncology Department and INSERM U1339/CNRS UMR 3666, Institut Curie, Paris, France; y Département d'Oncologie Médicale, Centre Léon Bérard, Lyon, France; z Department of Medical Oncology and Sarcoma Center, West German Cancer Center, University Hospital Essen, Essen, Germany

**Keywords:** primary pulmonary sarcoma, surgery, EURACAN, chemotherapy, radiotherapy, outcome, soft-tissue sarcoma

## Abstract

**Objective:**

To evaluate outcome and prognostic factors of patients with primary pulmonary sarcoma (PPS) who underwent curative-intent surgery within multimodality treatment.

**Methods:**

An international, multicenter, retrospective study including patients with PPS was performed through a network of sarcoma experts. Data on demographics, staging, treatment, and outcomes were retrieved. Overall survival was calculated from the date of diagnosis. Prognostic factors were assessed using uni- and multivariate analysis.

**Results:**

Eighteen centers from 9 countries contributed, for a total of 173 patients. One hundred fifteen patients (66%) underwent curative-intent surgery within multimodality treatment. There were 58 male patients (50%). Twenty-two patients (20%) had metastases, mainly to lung (n = 7, 30%) and pleura (n = 9, 39%). Thirty-three patients (30%) underwent preoperative chemotherapy. Extent of lung resection was sublobar (n = 11, 10%), lobar (n = 58, 54%), or bilobar/pneumonectomy (n = 39, 36%). Median tumor size was 85 mm. Sixty-nine patients had grade 3 tumors (71%). Resection was complete in 85 patients (75%). Lymphadenectomy was performed in 70 patients (63%), with nodal involvement in 10 (14%). Thirty-seven (37%) patients received adjuvant chemotherapy, and 27 (27%) patients received adjuvant radiotherapy. Overall survival was 49% and 31% at 5 and 10 years, respectively. Median follow-up was 33 months. Male gender (*P* = .003), age older than 60 years (*P* = .021), presence of metastasis (*P* = . 002), tumor size >40 mm (*P* = . 046), and incomplete resections (*P* = . 008) were independent prognostic factors for poor survival.

**Conclusions:**

In patients with curative-intent multimodal treatment for PPS, an encouraging 5-year survival rate of 49% can be achieved in expert centers. Independent prognostic factors may aid in selecting patients for curative treatment.

**Figure:**
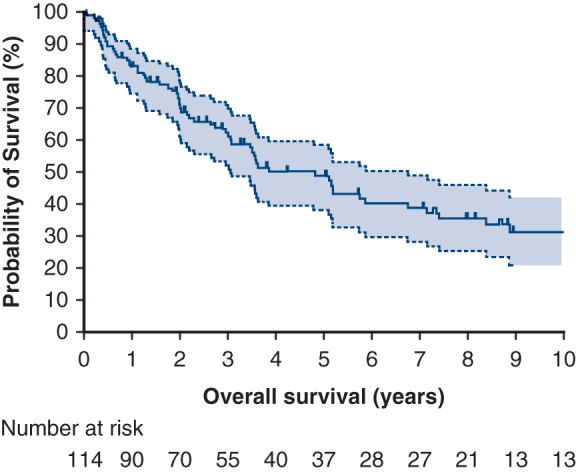
5-year survival for patients after surgery for primary pulmonary sarcoma was 49%.

**Central Message**

Age, gender, tumor size, presence of metastasis, and completeness of resection independently predict prognosis for resected patients with primary pulmonary sarcoma in a multicenter analysis.

**Perspective**

Identifying prognostic factors in this rare and highly heterogeneous population has enhanced our understanding of the natural history of primary pulmonary sarcoma. This knowledge may assist in selecting patients for perioperative multimodality therapy. Future collaboration between expert centers across continents could provide a sufficiently large population for conducting further research.

Primary pulmonary sarcoma (PPS) is an extremely rare type of soft-tissue sarcoma (STS) that originates in the lung. It accounts for less than 1.1% of all lung tumors and must be distinguished from the more common metastatic sarcomas that spread to the lung from extrapulmonary sites.^1^ Evidence on PPS is limited and primarily consists of retrospective case series with a small number of patients. PPS includes a heterogeneous group of tumors with various histologic subtypes, which complicates the development of standardized management protocols. In fact, more than 100 different histologic subtypes of soft-tissue tumors have been described according to the fifth edition of the World Health Organization's *Soft Tissue and Bone Tumours*.^2^ Currently, there are no established guidelines for the diagnosis and treatment of PPS. To optimize the management of this condition, we evaluated the outcomes and prognostic factors of patients with PPS who have undergone curative-intent surgery as part of a multimodal treatment approach.

## Methods

Members of the 2 relevant domains, “Thorax” and “Connective Tissue (Sarcomas),” within the European Reference Network on Rare Adult Solid Cancers (EURACAN), were contacted to gauge their interest in forming a multidisciplinary working group on thoracic sarcoma. This group would include medical oncologists, radiation oncologists, pathologists, radiologists, and thoracic surgeons. The working group on thoracic sarcoma was established on December 8, 2021. Data on demographics, diagnosis, staging, treatment, and outcomes were retrospectively analyzed for patients who underwent curative-intent surgery for PPS at participating institutions between 1995 and 2022. Patients with primary sarcomas of the pulmonary artery were excluded due to their unique clinical presentation and surgical therapy.

A specialized sarcoma pathologist (H-U.S.) reviewed the pathological diagnoses according to the fifth edition of the World Health Organization's *Soft Tissue and Bone Tumours*.^2^ Tumors from biopsy or surgical specimens were graded using the French Fédération Nationale de Centres de Lutte Contre le Cancer system.^3^

Survival was calculated from the date of diagnosis until death or the last follow-up. The cumulated survival rates were determined using Kaplan-Meier curves and compared with the log-rank test, and a hazard ratio (HR) was calculated using Cox regression. Statistically significant variables were further evaluated in a multivariate Cox regression model to identify independent prognostic factors. HRs were presented with 95% confidence intervals (CIs). Patients with missing data in 1 or more parameters were excluded from the regression analysis. Median values were presented along with their interquartile range (IQR). Statistical analyses were performed using IBM SPSS, version 29 (IBM Corp), and survival curves were generated with GraphPad Prism, version 10.4.1 for Mac (GraphPad Software). The study was approved by the institutional ethics committee, and patient consent was waived (19-8751-BO, July 30, 2019).

## Results

**Figure E1:**
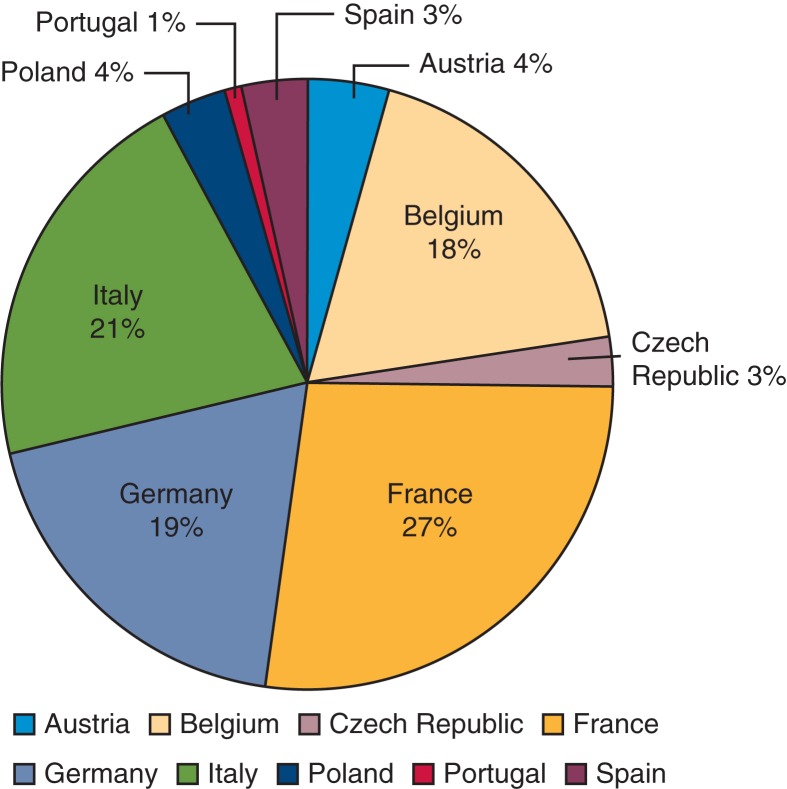
Distribution of patients, on the basis of the country of origin of the submitted data (N = 115).

**Figure E2:**
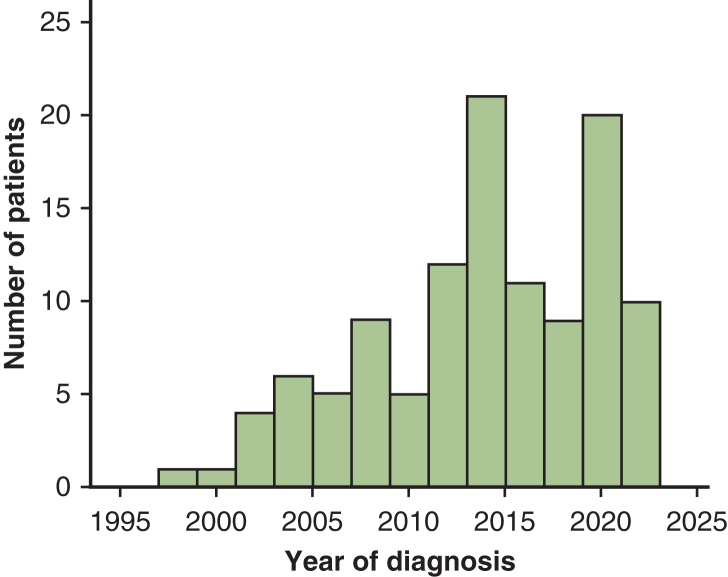
Distribution of patients according to their date of diagnosis.

A total of 173 patients with PPS from 18 centers from 9 European countries (Austria, Belgium, Czech Republic, France, Germany, Italy, Poland, Portugal, and Spain) were included. Of these, 115 patients (66%) underwent curative-intent surgery and represent the cohort of this study. Patient distribution, on the basis of the country of origin of submitted data is depicted in Figure E1. One half of the patients were diagnosed from 2014 (Figure E2). The median age was 56 years (IQR, 41-65 years). Fifty-eight patients were male (50%). A current or past smoking history was noted in 32 patients (28%). At the time of diagnosis, 88 patients (80%) were symptomatic, with symptoms including chest or back pain, dyspnea, cough, loss of appetite or weight, fatigue, and dysphonia. PPS was diagnosed during the work-up for pneumonia, hemothorax, or pleural effusion. Preoperative biopsy was performed in 86 patients (78%), using needle aspiration (n = 16, 19%), core needle biopsy (n = 40, 47%), or surgical biopsy (n = 30, 35%). The staging work-up was heterogeneous and included computed tomography (CT) scans of the chest, abdomen, and pelvis, as well as CT or magnetic resonance imaging of the brain, fluorodeoxyglucose positron emission tomography/CT, endobronchial ultrasound, or mediastinoscopy. Staging revealed metastatic disease in 23 patients (20%). The metastatic sites included the lungs (n = 7, 30%), pleura (n = 9, 39%), liver (n = 2, 9%), bone (n = 3, 13%), and other sites (n = 6, 26%). Patient distribution regarding the administration of perioperative chemotherapy and radiotherapy is summarized in Table 1. If pre- or postoperative treatment was administered, chemotherapy was more commonly used than radiation. The chemotherapy regimen consisted mainly of a combination of doxorubicin and ifosfamide.

**Table 1:** Distribution of patients who received perioperative chemotherapy and radiotherapy

Covariates	Postoperative chemotherapy		Postoperative radiotherapy	
	Yes	No	Yes	No
Preoperative chemotherapy				
Yes	8 (7%)∗	25 (23%)∗	9 (9%)†	24 (24%)†
No	29 (27%)∗	46 (43%)∗	18 (18%)†	49 (49%)†
Preoperative radiotherapy				
Yes	1 (1%)‡	6 (6%)‡	2 (2%)§	5 (5%)§
No	34 (32%)‡	64 (61%)‡	25 (25%)§	69 (68%)§

Where N is the number of patients available for the analysis.

∗ N = 108.

† N = 100.

‡ N = 105.

§ N = 101.

**Figure 1:**
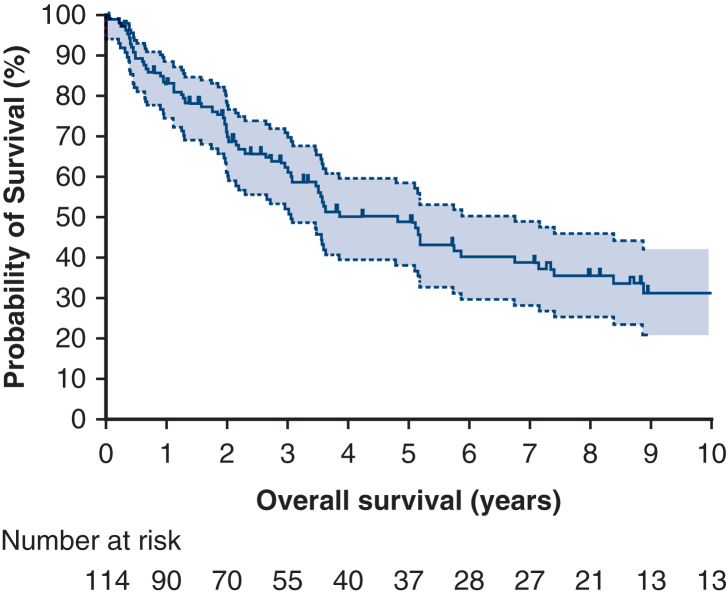
Overall survival for the whole cohort. The 95% confidence intervals are represented by the *light gray zone*.

Lung resections were categorized as follows: sublobar (n = 11, 10%), lobar (n = 58, 54%), and bilobar/pneumonectomy (n = 39, 36%). Lymphadenectomy was performed in 70 patients, accounting for 63% of the cases. Among these, 10 patients (14%) had positive lymph nodes identified on pathology, which included hilar lymph nodes (n = 7, 70%) and mediastinal lymph nodes (n = 5, 50%). Thirty-day and 90-day mortality were 0.8% and 1.7%, respectively. The median tumor size was 85 mm (IQR, 47-130 mm), whereas the mean tumor size was 95 mm. A total of 26 histologic subtypes were observed, with synovial sarcoma being the most common (n = 49, 44%). This was followed by leiomyosarcoma (n = 11, 10%), Ewing sarcoma (n = 10, 9%), angiosarcoma (n = 6, 5%), sarcoma not otherwise specified (n = 5, 5%), undifferentiated pleomorphic sarcoma (n = 5, 5%), malignant peripheral nerve sheath tumor (n = 4, 4%), inflammatory myofibroblastic tumor (n = 2, 2%), epithelioid hemangioendothelioma (n = 2, 2%), and other subtypes (n = 17, 15%). Most tumors were classified as grade 3 (n = 69, 71%), followed by grade 2 (n = 19, 20%) and grade 1 (n = 9, 9%). Complete microscopic resection (R0) was achieved in 85 patients (75%). Incomplete resections were noted in 28 patients, which included incomplete microscopic resections (R1, n = 14, 12%) and macroscopic resections (R2, n = 14, 12%). Patients with symptoms at time of diagnosis had not a statistically significant different rate of complete resection than patients who were asymptomatic (*P* = .068, Pearson χ^2^). Figure 1 shows the overall survival rates for patients who underwent curative-intent surgery for PPS. The survival rates were 49% at 5 years and 31% at 10 years. The median follow-up period for these patients was 33 months. Details of the univariate and multivariate analyses for overall survival can be found in Table 2.

**Table 2:** Results of the univariate and multivariate analysis

Covariates	Univariate analysis		Multivariate analysis	
	HR (95% CI)	*P* value	HR (95% CI)	*P* value
Age, y				
≤40	Reference		Reference	
41-60	1.14 (0.59-2.17)	.690	1.42 (0.70-2.88)	.331
>60	1.77 (0.94-3.33)	.080	2.31 (1.14-4.68)	.021
Gender				
Female	Reference		Reference	
Male	2.16 (1.30-3.58)	.003	2.25 (1.31-3.87)	.003
Tumor size				
≤40	Reference		Reference	
>40	3.33 (1.44-7.76)	.005	2.43 (1.02-5.79)	.046
Metastasis				
No	Reference		Reference	
Yes	2.57 (1.45-4.53)	.001	2.81 (1.47-5.37)	.002
Resection				
Complete	Reference		Reference	
Incomplete	2.41 (1.43-4.05)	.001	2.14 (1.22-3.77)	.008
Symptoms at diagnosis				
No	Reference			
Yes	1.63 (0.87-3.05)	.131		
Tumor side				
Right	Reference			
Left	0.77 (0.48-1.26)	.303		
Preoperative chemotherapy				
No	Reference			
Yes	1.02 (0.81-1.72)	.935		
Postoperative chemotherapy				
No	Reference			
Yes	1.14 (0.69-1.90)	.614		
Preoperative radiotherapy				
No	Reference			
Yes	0.80 (0.32-2.00)	.632		
Postoperative radiotherapy				
No	Reference			
Yes	0.92 (0.52-1.63)	.775		
Lymph node removal				
No	Reference			
Yes	0.79 (0.48-1.29)	.340		
pN				
0	Reference			
Positive	1.02 (0.40-2.59)	.964		
Tumor grade				
1	Reference			
2-3	2.94 (0.72-12.03)	.133		
Smoker				
No	Reference			
Yes	1.32 (0.79-2.19)	.294		
Type of lung resection				
Wedge	Reference			
Lobar	1.47 (0.51-4.17)	.475		
Bilobar/pneumonectomy	3.07 (1.07-8.77)	.036		
Synovial sarcoma				
No	Reference			
Yes	1.22 (0.76-1.98)	.415		

*HR*, Hazard ratio; *CI*, confidence interval.

**Figure 2:**
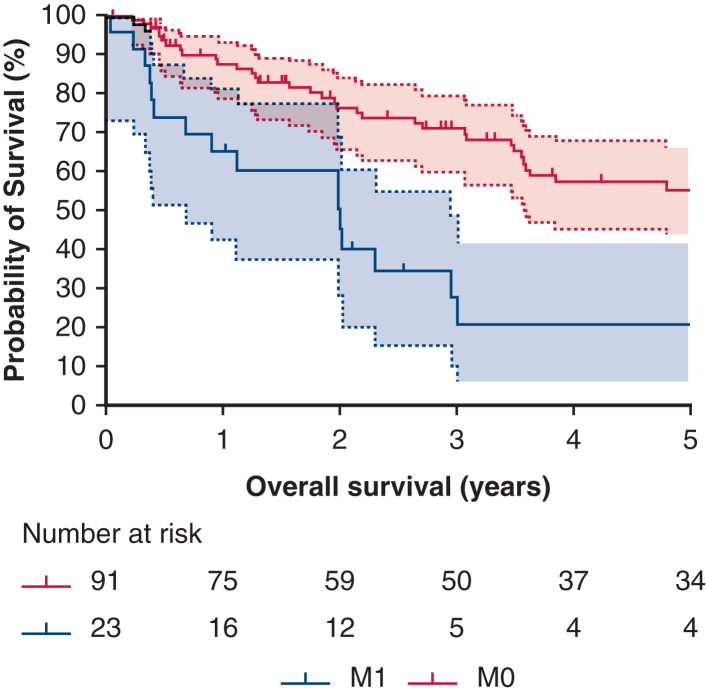
Survival depending on the presence of metastasis at diagnosis. The 95% confidence intervals for both curves are represented by the *light red* and *blue zones*.

**Figure 3:**
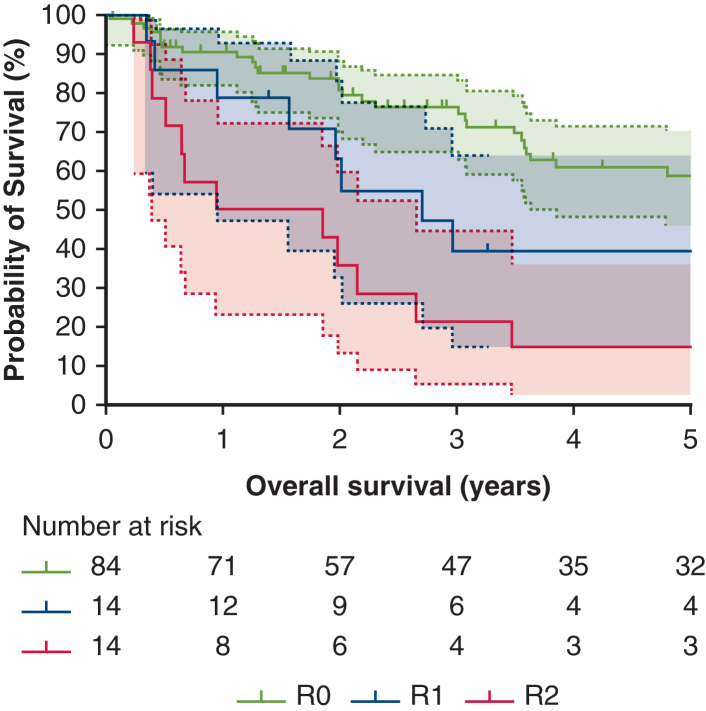
Survival depending on completeness of resection. The 95% confidence intervals for the 3 curves are represented by the *light gray*, *blue*, and *red zones*. *R0*, Complete resection; *R1*, incomplete microscopic resection; *R2*, incomplete macroscopic resection.

Six parameters—age, gender, tumor size, presence of metastasis at diagnosis, completeness of resection, and type of lung resection—were found to be statistically significant in the univariate analysis. As a result, these parameters were included in the multivariate analysis. In this analysis, the type of resection lost its significance (*P* = .347; HR, 1.73; CI, 95% 0.55-5.38), whereas the other 5 parameters were identified as independent prognostic factors. Survival curves for patients with metastasis at diagnosis are shown in Figure 2. Patients with metastasis at diagnosis exhibited significantly worse outcomes compared with those without metastasis (*P* = .001). The median survival times were 5.18 years (95% CI, 3.98-6.39) for patients without metastasis and 2.01 years (95% CI, 1.98-2.05) for those with metastasis. Figure 3 illustrates the survival curves on the basis of the completeness of resection. There were statistically significant differences in survival among the various types of completeness of resections (*P* < .001). The median survival times were 5.87 years (95% CI, 3.78-7.99), 2.71 years (95% CI, 1.09-4.34), and 0.95 years (95% CI, 0.00-3.09), for R0, R1, and R2, respectively.

## Discussion

Because of the rarity of PPS, data in the literature are scarce and primarily on the basis of case reports or small series of fewer than 25 surgically treated patients. Two larger studies reported outcomes for 31 and 48 patients after surgery for PPS.^4^^,^^5^ To better understand clinical outcomes in patients with PPS, authors conducted analyses using the Surveillance, Epidemiology, and End Results (SEER) database, evaluating 326 and 80 patients from the periods 1988-2008 and 2010-2019, respectively.^6^^,^^7^ In another work, 515 patients both treated with and without surgery were retrieved from the SEER database from 1998 to 2015.^8^ A nomogram was built on the basis of age, histology type, tumor size, presence of lymph node resection, “summary stage” and tumor differentiation. The nomogram was used to divide patients into high and low-risk groups for death to further evaluate the benefit of chemotherapy and radiation in both groups. Despite the large size of these patient cohorts, data from the SEER database have notable limitations, including unrecorded variables, underreported and incomplete adjuvant treatment data, and inconsistencies in coding and reporting.^7^ In the study by Spraker and colleagues,^6^ details were unavailable regarding the use of adjuvant chemotherapy, radiotherapy dosage, or completeness of resection. In the study of Gu and colleagues,^8^ the lack of precise definition for “summary stage” renders the nomogram poorly applicable. In the current study, 18 centers from 2 specific EURACAN domains contributed data, allowing the evaluation of 115 patients with surgically treated PPS. EURACAN is one of the 24 European Reference Networks funded by the European Commission. It provides care for patients with rare adult solid cancers and comprises 102 highly specialized centers across 25 European countries.^9^ In our large cohort, the 5-year overall survival rate was 49%, aligning with most published studies, which report survival rates ranging from 38% to 50%.^5^^,^^6^^,^^10^, ^11^, ^12^, ^13^ In contrast, a smaller single-center study of 23 surgically resected patients reported 3- and 5-year survival rates of 69%.^14^

In our cohort, age, gender, tumor size, presence of metastasis, and completeness of resection were identified as five independent factors impacting survival. Patients with tumors larger than 40 mm had a 2.4 times greater risk of dying compared to those with tumors measuring 40 mm or less. These findings are consistent with previous research, as Spraker and colleagues^6^ reported similar results using a tumor size cutoff of 50 mm. In addition, our results confirm findings from univariate analyses conducted by other authors.^15^^,^^16^ Tumor resectability remains a strong determinant of survival. Patients with unresectable disease who underwent radiotherapy alone had worse survival outcomes compared to those who underwent surgery alone (HR, 2.6; 95% CI, 1.76-3.88).^6^ The primary goal of surgery is complete tumor resection with negative microscopic margins, as incomplete resection significantly impairs survival.^6^^,^^11^^,^^14^^,^^16^ In our study, patients who underwent incomplete resection had a 2.1 times greater risk of dying compared with those who had a complete resection. Therefore, we strongly advise that an experienced surgeon thoroughly evaluate imaging to best select patients deemed resectable. For STS, histologic grading is recognized as the most important prognostic factor, predicting both distant metastasis and disease-specific survival.^17^^,^^18^ Although some studies have reported similar findings for PPS, we did not observe any influence of tumor grading on survival in our cohort.^6^^,^^10^ The reason for this discrepancy remains unclear. However, it is important to note that grading is neither histology-specific nor site/organ-specific and should always be interpreted within the broader context of the disease process.^18^ Some authors have demonstrated the prognostic relevance of grading in specific histologic subtypes, such as malignant fibrous histiocytoma (renamed undifferentiated pleomorphic sarcoma not otherwise specified in 2002), unclassified sarcoma, synovial sarcoma, leiomyosarcoma, and liposarcoma. However, grading did not hold prognostic significance in pediatric rhabdomyosarcoma.^17^^,^^19^ To further explore this, we specifically analyzed the synovial sarcoma subpopulation to assess whether grading influenced survival. No significant difference in survival was observed between patients with G1 and G2-3 tumors (data not shown). In retroperitoneal STS, grading becomes a prognostic factor only after complete tumor resection, as patient outcomes primarily depend on achieving an R0 resection.^18^^,^^20^ In our cohort, no survival difference was found between patients with G1 and G2-3 tumors when analyzing only those who underwent complete (R0) resection (data not shown). A trend toward improved survival for G1 versus G2-3 tumors was noted in patients who received preoperative chemotherapy or radiotherapy (data not shown). This suggests that perioperative treatment may have mitigated the adverse impact of higher tumor grades on survival. Patients who underwent pre- or postoperative chemotherapy alone, pre- or postoperative radiotherapy alone, or combined pre- and postoperative chemotherapy and/or radiotherapy did not show differences in survival compared to those treated with surgery alone. Another potential explanation for our findings is the method of grade assessment. In our study, tumor grading may have been on the basis of the surgical specimen after preoperative treatment rather than the standard approach of grading only untreated primary sarcomas. Indeed, our database did not specify whether grading was assessed on a pretreatment biopsy or on the operative specimen.

There is no evidence regarding the use of perioperative chemotherapy or radiotherapy in PPS. Current treatment recommendations are determined from evidence from the management of STS of the trunk and extremities. The benefit of pre- or postoperative chemotherapy in extremity STS remains uncertain, and it is therefore not considered standard therapy for patients with localized STS of the extremities. However, it is generally accepted that the decision to administer pre- or postoperative chemotherapy should be discussed in a multidisciplinary sarcoma tumor board. It is likely best reserved for patients with chemotherapy-sensitive histologic subtypes, such as myxoid liposarcoma or synovial sarcoma, as well as for those at high risk of mortality from STS, specifically patients with large and high-grade tumors.^18^^,^^21^, ^22^, ^23^ Radiotherapy is known to enhance local control in STS of the extremities and trunk and is typically recommended for high-grade (G2-G3) tumors.^18^^,^^24^ Multiple studies have evaluated the role of pre- and postoperative radiotherapy without demonstrating the superiority of one approach over the other.^18^ In the study by Spraker and colleagues,^6^ patients who underwent surgery plus radiotherapy showed a trend toward worse survival compared to those who had surgery alone. However, this was likely due to selection bias, as patients in the surgery-plus-radiotherapy group had a greater-risk profile, with more tumors larger than 5 cm (65% vs 51%), more high-grade tumors (94% vs 70%), and a greater incidence of node-positive disease (27% vs 10%). The addition of adjuvant radiotherapy did not fully compensate for the survival disadvantage associated with these greater-risk tumor characteristics in the surgery-plus-radiotherapy group.

Our study represents the largest analysis of surgical outcomes in PPS patients, including data on perioperative chemotherapy and radiotherapy. However, it is important to acknowledge the limitations inherent to its retrospective design. The long study period is partially mitigated by a high rate of diagnosis from 2014. In addition, our data lacks information on whether tumor grading was assessed from biopsy or the surgical specimen. Last but not least, the generalizability of our findings may be limited, as patients were treated within an expert sarcoma center network.

Among patients undergoing curative-intent surgery and multimodal treatment for PPS, an encouraging 5-year survival rate of 49% was achieved in expert centers. Independent prognostic factors—such as age, gender, tumor size, presence of metastasis, and completeness of resection were identified. These factors may help guide patient selection for curative treatment. The rarity and heterogeneity of PPS, with its diverse histological subtypes and variable responses to therapy, continue to pose challenges in generating high-quality evidence and conducting prospective studies. To overcome these limitations, the establishment of a global network of specialized centers should be encouraged, similar to the Trans-Atlantic Australasian Retroperitoneal Sarcoma Working Group.^25^ Such an initiative could facilitate the inclusion of a sufficiently large patient population to support clinically meaningful research and potentially lead to the creation of an international prospective PPS registry.

### Webcast

You can watch a Webcast of this AATS meeting presentation by going to: https://www.aats.org/resources/outcome-of-patients-with-curat-9481.

**Figure:**
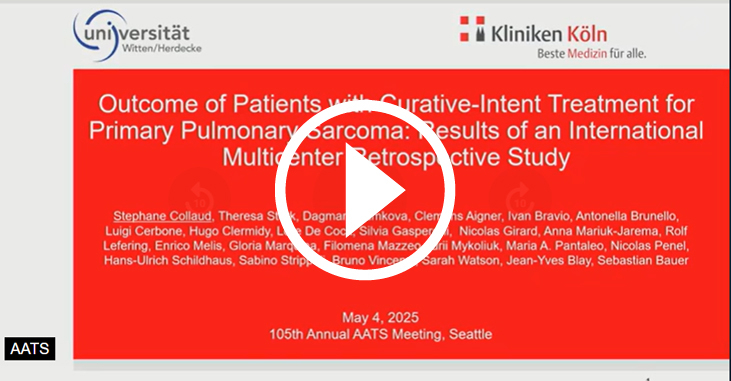

## Conflict of Interest Statement

A.B. reports honoraria from Boehringer Ingelheim, Deciphera, GSK for consultancy or participation to advisory boards; and from Pharmamar for travel and accommodations expenses. S.C. reports financial relationships as a speaker for AstraZeneca and MedXpert, as a consultant/advisor for Materialise and Novocure, and for research support from Materialise. All other authors reported no conflicts of interest.

The *Journal* policy requires editors and reviewers to disclose conflicts of interest and to decline handling or reviewing manuscripts for which they may have a conflict of interest. The editors and reviewers of this article have no conflicts of interest.

## References

[1] Collaud S., Stork T., Schildhaus H.U. (2020). Multimodality treatment including surgery for primary pulmonary sarcoma: size does matter. J Surg Oncol.

[2] Sbaraglia M., Bellan E., Dei Tos A.P. (2021). The 2020 WHO Classification of. Soft Tissue Tumours: news and perspectives. Pathologica.

[3] Trojani M., Contesso G., Coindre J.M. (1984). Soft-tissue sarcomas of adults; study of pathological prognostic variables and definition of a histopathological grading system. Int J Cancer.

[4] Robinson L.A., Babacan N.A., Tanvetyanon T., Henderson-Jackson E., Bui M.M., Druta M. (2021). Results of treating primary pulmonary sarcomas and pulmonary carcinosarcomas. J Thorac Cardiovasc Surg.

[5] Petrov D.B., Vlassov V.I., Kalaydjiev G.T. (2003). Primary pulmonary sarcomas and carcinosarcomas—postoperative results and comparative survival analysis. Eur J Cardiothorac Surg.

[6] Spraker M.B., Bair E., Bair R., Connell P.P., Mahmood U., Koshy M. (2013). An analysis of patient characteristics and clinical outcomes in primary pulmonary sarcoma. J Thorac Oncol.

[7] Huang Q., Li W., He X. (2023). Prognostic visualization model for primary pulmonary sarcoma: a SEER-based study. Sci Rep.

[8] Gu H., Song R., Beeraka N.M. (2023). SEER-based survival nomogram (1998-2015) based on “stage, lymph node dissection, tumor size and degree of differentiation, and therapies” for prognosis of primary pulmonary sarcoma. Technol Cancer Res Treat.

[9] EURACAN Mission and goals. https://www.euracan.eu/about-us/about-euracan.

[10] Janssen J.P., Mulder J.J.S., Wagenaar S.S., Elbers H.R.J., van den Bosch J.M.M. (1994). Primary sarcoma of the lung: a clinical study with long-term follow-up. Ann Thorac Surg.

[11] Regnard J.F., Icard P., Guibert L., de Montpreville V.T., Magdeleinat P., Levasseur P. (1999). Prognostic factors and results after surgical treatment of primary sarcomas of the lung. Ann Thorac Surg.

[12] Porte H.L., Metois D.G., Leroy X., Conti M., Gosselin B., Wurtz A. (2000). Surgical treatment of primary sarcoma of the lung. Eur J Cardiothorac Surg.

[13] Etienne-Mastroianni B., Falchero L., Chalabreysse L. (2002). Primary sarcomas of the lung: a clinicopathologic study of 12 cases. Lung Cancer.

[14] Bacha E.A., Wright C.D., Grillo H.C. (1999). Surgical treatment of primary pulmonary sarcomas. Eur J Cardiothorac Surg.

[15] Collaud S., Stork T., Adámková Krákorová D. (2024). 1732P Primary pulmonary sarcoma: a EURACAN project. Ann Oncol.

[16] Yamada Y., Kaplan T., Soltermann A. (2021). Surgical outcomes and risk analysis of primary pulmonary sarcoma. Thorac Cardiovasc Surg.

[17] Coindre J.M., Terrier P., Guillou L. (2001). Predictive value of grade for metastasis development in the main histologic types of adult soft tissue sarcomas: a study of 1240 patients from the French Federation of Cancer Centers Sarcoma Group. Cancer.

[18] Gamboa A.C., Gronchi A., Cardona K. (2020). Soft-tissue sarcoma in adults: an update on the current state of histiotype-specific management in an era of personalized medicine. CA Cancer J Clin.

[19] Hashimoto H., Daimaru Y., Takeshita S., Tsuneyoshi M., Enjoji M. (1992). Prognostic significance of histologic parameters of soft tissue sarcomas. Cancer.

[20] Lewis J.J., Leung D., Woodruff J.M., Brennan M.F. (1998). Retroperitoneal soft-tissue sarcoma: analysis of 500 patients treated and followed at a single institution. Ann Surg.

[21] Gronchi A., Miah A.B., Dei Tos A.P. (2021). Soft tissue and visceral sarcomas: ESMO-EURACAN-GENTURIS Clinical Practice Guidelines for diagnosis, treatment and follow-up(∗). Ann Oncol.

[22] Pasquali S., Pizzamiglio S., Touati N. (2019). The impact of chemotherapy on survival of patients with extremity and trunk wall soft tissue sarcoma: revisiting the results of the EORTC-STBSG 62931 randomised trial. Eur J Cancer.

[23] Callegaro D., Miceli R., Bonvalot S. (2018). Impact of perioperative chemotherapy and radiotherapy in patients with primary extremity soft tissue sarcoma: retrospective analysis across major histological subtypes and major reference centres. Eur J Cancer.

[24] von Mehren M., Randall R.L., Benjamin R.S. (2018). Soft tissue sarcoma, version 2.2018, NCCN clinical practice guidelines in oncology. J Natl Compr Canc Netw.

[25] Transatlantic Australasian Retroperitoneal Sarcoma Working Group. https://tarpswg.org.

